# Book Reviews

**Published:** 1913-01

**Authors:** 


### BOOK REVIEWS

Polk's Medical Register and Directory of North America, 1912. R. L. Polk & Co., publishers, Detroit. 2378 pages, $10.00.

The present, twelfth edition, maintains its standard of convenience and accuracy. Beside a listing of physicians by localities and alphabetically for the U. S. and Canada, it includes lists of medical schools, journals, sanitariums, hospitals, societies, etc., a digest of medical laws for each state and various other valuable information almost indispensable to any physician in touch with professional matters. We have prepared the following table at some pains, as a basis for the study of various economic problems.

| State | Population 1910 | Number of Physicians | Ratio (Average Medical Clintele) |
|---|---|---|---|
| Alabama | 2,138,093 | 2,303 | 928 |
| Alaska | 64,356 | 81 | 794 |
| Arizona | 204,354 | 260 | 786 |
| Arkansas | 1,574449 | 1,952 | 801 |
| California | 2,377,549 | 4,425 | 537 |
| Colorado | 799,024 | 1,593 | 501 |
| Connecticut | 1,114,756 | 1,457 | 765 |
| Delaware | 202,322 | 256 | 790 |
| District of Columbia | 331,069 | 1,027 | 322 |
| Florida | 752,619 | 996 | 756 |
| Georgia | 2,609,121 | 2,669 | 977 |
| Idaho | 325,594 | 400 | 814 |
| Illinois | 5,638,591 | 9,679 | 583 |
| Indiana | 2,700,876 | 4,913 | 550 |
| Iowa | 2,224,771 | 3,604 | 617 |
| Kansas | 1,690,949 | 2,798 | 604 |
| Kentucky | 2,289,905 | 3,309 | 692 |
| Louisiana | 1,656,385 | 1,860 | 890 |
| Maine | 742,371 | 1,116 | 665 |
| Maryland | 1,294,450 | 2,078 | 623 |

| | | | |
|---|---|---|---|
| Massachusetts | 3,366,416 | 5,285 | 637 |
| Michigan | 2,810,173 | 4,102 | 685 |
| Minnesota | 2,075,708 | 2,094 | 991 |
| Mississippi | 1,797,114 | 1,646 | 1092 |
| Missouri | 3,293,335 | 6,136 | 536 |
| Montana | 376,053 | 494 | 761 |
| Nebraska | 1,192,214 | 1,697 | 702 |
| Nevada | 81,875 | 130 | 629 |
| New Hampshire | 430,572 | 685 | 628 |
| New Jersey | 2,537,167 | 2,572 | 986 |
| New Mexico | 327,396 | 408 | 802 |
| New York | 9,113,614 | 13,977 | 652 |
| North Carolina | 2,206,287 | 1,831 | 1205 |
| North Dakota | 577,056 | 579 | 996 |
| Ohio | 4,767,121 | 7,398 | 659 |
| Oklahoma | 1,657,155 | 2,168 | 764 |
| Oregon | 672,765 | 937 | 718 |
| Pennsylvania | 7,665,111 | 10,648 | 719 |
| Rhode Island | 542,610 | 726 | 747 |
| South Carolina | 1,515,400 | 1,258 | 1204 |
| South Dakota | 583,888 | 674 | 866 |
| Tennessee | 2,184,789 | 2,790 | 783 |
| Texas | 3,896,542 | 5,126 | 760 |
| Utah | 373,351 | 422 | 884 |
| Vermont | 355,956 | 671 | 530 |
| Virginia | 2,061,612 | 2,173 | 948 |
| Washington | 1,141,990 | 1,533 | 745 |
| West Virginia | 1,221,119 | 1,627 | 768 |
| Wisconsin | 2,333,860 | 2,352 | 992 |
| Wyoming | 145,965 | 168 | 868 |
| Total | 91,972,267 | 129,002 | 713 |

Continental U. S. (Exclusive of Alaska).

These statistics seem to show a much more favorable economic relation of profession to population than has been supposed. although they do not alter the actual experience of over-supply of physicians. We would be glad to have our calculation of ratios verified or corrected as, in running through a table of logarithms, a blur in the print or an error in following a line, makes a considerable error . Unusual ratios, however, have been verified by long division and we are certain that any mistakes will be within limits which will not affect the general deductions. It has been believed that the present directory would show over 140,00 physicians and that the profession would have shown an increase over previous listings. On the contrary, in many states, there has been an actual diminution. With increasing competition in directories, more systematic canvass by local organizations, expedition of enumeration by more careful city directories and larger telephone sevrice, and the carrying out of a constant revision during the intervening time, it seems certain however, that the error due to accidental omissions has decreased. We have elsewhere called attention to the probable increase in professional mortality by the fact that the present requirements as to medical education and licensure, have increased the average age at which practice is begun, while the actual decline in the total of medical graduates, still further increases the average age of the profession. Hence, it must have a higher mortality than previously, subject to the general effect of sanitary and hygienic improvements. Again, the Civil War not only called for a relatively large medical profession for army service, but, at its close, left an unusually large number of unemployed men in the twenties and thirties who naturally drifted in medicine. Within the last few years, there has been an enormous death list of physicians who had served in the Civil War, as surgeons or otherwise.

As to whether Polk's statistics are low, we can decide only by reference to Buffalo, with the conditions in which city we are reasonably familiar. Polk states that there are 750 physicians in Buffalo. The State Directory compiled with as great care as possible, gives only 703. Of these, we know personally, 42 that are either dead, moved away or engaged in other occupations, and only 4 additions. Many of the listing in either book, are retired or are internes not yet in private practice. Counting physicians actually practicing, Polk's figures are probably 100 too high for Buffalo. It is not probable that the listing for the entire country is correspondingly high and that 20% should be substracted from the whole number but it is not at all improbable that the active list should be reduced by 10% making the entire practicing profession not far from 115,000, corresponding to an average clientele of almost exactly 800.

There is still another factor to be considered: The population of the U. S. is increasing at a rate of slightly over 20% in a decade. Even allowing for the fact that this represents an analogue to compound interest, the yearly increase is nearly 2%. It is a conservative estimate to place the present population at 95 million, which further increases the average clientiele to 826.

Within the next few years, it is practically impossible that the average graduation of medical students can exceed 4000 a year (See page 159, October issue). The annual mortality of the 129,000 physicians will amount to about 2700. The various public services, teaching, laboratory work, etc., will demand at least 400 (10%) of the graduates. For the remaining 900—a liberal estimate—there is annual increment in population of about 1,800,-000, perhaps more. Approximately, the average clientele can be expected to increase by about 10 a year. By concerted effort, mainly by increasing low matriculation requirements to those of higher standard already in force in many states, it is feasible to bring about, within a very few years, a reduced graduation that will simply make good the death rate and demand of the public services. In this way, the average clientele could be increased by about 15 a year, or to 950 in 1920.

The irregular distribution of population and physicians is noteworthy, the average clientele ranging from a minimum of 322 for the District of Columbia—obviously fictitiously low on account of the considerable number of government surgeons stationed at Washington—to 1205 for North Carolina. There is generally, a high clientele in the states where sparse distribution of population demands the opposite condition, although the figures may possibly be due to difficulties in getting accurate statistics of physicians. The attraction of large educational centers is also conspicuous, and the excess probably consists largely of laboratory workers, teachers not in practice, and internes. The call of certain states to the tuberculosis and otherwise invalid members of the profession is also apparent.

---

THE PRACTICAL MEDICINE SERIES. Vol. VII, Pediatrics by Dr. Isaac A. Abt and Dr. May Mitchell of Chicago; Orthopaedic Surgery by Drs. John Ridlon and Charles A. Parker of Chicago. Published by the Year Book Publishers, Chicago. 240 pages, $1.25 (Price for the Series of 10 volumes, $10).

This is an excellent review of literature, of handy size, already familiar to our readers.

---

PROGRESSIVE MEDICINE. (quarterly), Vol. 14, No. 4, Dec., 1912. This series, under the editorship of Drs. H. A. Hare and L. F. Appleman of Philadelphia, is well known. The present number covers the alimentary tract, kidneys, genito-urinary organs, various surgical topics and contains a practical therapeutic referendum. Price, $6.00 per annum.

BUILDING A PROFITABLE PRACTICE, by Dr. Thomas F. Reilly of New York, published by the J. B. Lippincott Co., Philadelphia, 290 pages, price not stated.

The book urges experience as interne and warns against the common cephalomegaly, gives practical information as to post graduate study, at home and abroad, state license, opportunities in the public service, etc. It advises the young doctor on almost every conceivable point, such as the equipment and location of an office, the fees to be charged, warns him against contract practice, vivisects consultants so keenly that they will feel no pain, and, in short, acts as a mentor till one has acquired necessary experience and has made his place in the profession. The advice is thoroughly ethical, but shrewdly tears away the ethical mask of greed and graft, from those who would exploit the inexperienced physician.

---

THE CHEMIC PROBLEM IN NUTRITION. Dr. John Aulde of Philadelhia, published by the author. 410 pages, $3.00.

The work deals with magnesium infiltration, as a new disease or, rather as one newly discovered in its relations to a wide variety of clinical conditions. General discussions of nutrition, metabolism, germination, and dietetics precede the detailed discussion of diseases of systems and symptomatic groups. The work is iconoclastic in many respects and sweeping in its claims for eliminant treatment of a peculiar kind. We do not venture to support the distinguished author but we ask for him a thorough study of his treatise and a fair trial of his methods.

---

THE PSYCHOLOGY OF INSANITY. Dr. Bernard Hart, London, published by the Cambridge University Press; G. P. Putnam's Sons, New York. (Series of Cambridge Manuals) 40 cents. 175 pages.

In the review of the history, it is stated that Hippocrates first discarded the daemonologic theory and clearly recognized insanity as a lesion of the brain. The analogy of the disordered psychic state of the insane to various inconsistencies and tendencies in the normal individual, is clearly traced and the whole discussion is lucid and interesting.

---

A PRACTICAL MEDICAL DICTIONARY, Thomas Lathrop Stedman, A. M., M. D., New York, published by William Wood & Co., New York. 1025 pages, profusely illustrated, 2d edition, limp leather, $4.50, plain, $5.00 with thumb idex.

The exhaustion of a large edition in a year speaks well for this work and has allowed still further improvement. Every detail of the publisher's art has been used to lighten weight and facilitate reference, in spite of the enormous scope of the work. Beside its avowed function, the book contains much biographic information, presents many useful tables and gives hints on various practical points, wherever these could be presented without too great excursion from the province of a dictionary. The author is scholarly, yet does not insist too strongly on reforms of nomenclature, hinting instead at the proper substitutes for incorrect terms in common use.

---

ANUARIO MEDICO SUD AMERICANO, 1912. Puccio y Muhlrad, 450 Calle Maipu, Buenos Aires. 394 pages, paper cover, $5.00.

This gives lists of physicians, druggists, dentists, etc., of Argentina, Uruguay and Chili. Among the lists of specialists of the

"Federal Capital," are over 200 surgeons, 38 gastro-enterologists and others in proportion. As we understand it, the term "Federal Capital" refers to a territory like the District of Columbia, including the city of Buenos Aires. While the names are mainly Spanish, there is obviously a considerable mingling of other races.

---

Mortality Statistics, 1909, issued by the Bureau of the Census. 1912.

This is a valuable volume for reference, although we are more anxious to receive the Census Reports of 1910 which, we understand, have been delayed like the present volume, not through any fault of the Bureau, but on account of lack of appropriations for hastening the work. Still, as the estimates of population have been corrected in accordance with facts from the census of 1910, this report is of more practical value than might be supposed. We select the following points of interest at random. The registration area now includes 56.1% of the total population: 50,870,518 of 90,691,354 total estimated population for 1909. In a thousand deaths, 539.5 are of males, 460.5 of females, the difference being due to the almost universal excess of male births and the still inevitable excess in the first year of life. The relative deaths by age (not mortality for population at any age but proportion of a mixed series of deaths) drop rapidly to 8.6 for the age of 4, reach a minimum of 15.9 for the five year period 10—14, and increase gradually to 58.5 for the period 65—69. 1.8 per thousand deaths occur within the ages 95-99 and 0.4 after 100.

---

A Century of Population Growth in the U. S. 1790-1900.

This is a most interesting volume prepared by the Bureau of the Census. We select a few topics at random.

The standard of 8000 population as the minimum of "Urban Population" was first reached by Boston between 1700 and 1710. In 1790, when the first U. S. Census was taken, there were only five cities of greater population: 1. Philadelphia, (42,444), 2. New York (33,131), 3. Boston (18,038), 4. Charleston (16,359), 5. Baltimore (13,503). Salem, however, was at nearly the urban minimum (7,921). In 1730, nearly 5% of the total population of what is now the U. S., was urban in this sense. Owing to the relative insignificance of industrial and commercial occupations, the urban population declined (relatively to the total), till 1780 and then rose gradually to reach nearly the 5% ratio in 1810 and 1820. Since 1820, it has always increased and at nearly the same rate except for a slight diminution in the decade from 1870 to 1880. In 1900, the urban population amounted to 40.5%

and, in 1910 to 46.3% of the total. By the next census, it will almost certainly have passed 50%.

The matter of fertility is important, medically and sociologically. In 1790, there were eight white adults over 20 to 10 children. In 1900, there were 16 to ten. This is nearly the same ratio as for most European countries except France, where the ratio is 24 to 10. In various other ways, the fertility is shown to be less.

The 3,929,214 inhabitants of the U. S. in 1790 were divided among about 30,000 sur-names. Smith, which was a very rare name in America up 1700, now composed nearly one per cent. Eight other names comprised about 3% more. Members of colonial families will find statistics of a number of heads of families and others, of their surname, alphabetically tabulated.

Of the approximately 75 millions in 1900, it is estimated that 35 millions are American according to George Francis Train's definition—to which Carl Schurtz objected that G. F. T.'s Americans were so by a series of accidents of birth while he himself was an American by intelligent and deliberate choice. We are rather inclined to regard this estimate as high and that not more than a third of all Americans are of colonial stock. For most cities, the proportion is much lower. Nearly nine million of the population in 1900 were "colored," mostly negroes. Practically all the negroes are colonials and, that the race question is being solved by natural means, is indicated that, if relatively as numerous as in 1790, they would be more than one and a half times their present number.

From the preliminary Bulletin of the Census of 1910, we glean that the population has always increased absolutely from decade, to nearly 92 millions in 1910; that the increase for a decade has always been greater than for the previous decade, except for that covering the Civil War, but that the percentage increase which kept close to 35% between 1800 and 1860 has fallen to about 21% for each of the last two decades.

The population per square mile has increased from 4.5 in 1790 to 31 in 1910. Aside from the District of Columbia, ten states including New York as fifth in order, have populations of over 100 per square mile. Excluding the District of Columbia (5518 per square mile) and Rhode Island (508) which are virtually cities with suburbs, the most densly populated states are Massachusetts (418) and New Jersey (337). Porto Rico comes next with 325. If the whole continental U. S. were populated to the degree of a little over 300 per square mile, which seems compatible with self-support, the total population would be about a billion. This number would be reached at the present rate in about A. D. 2030 or 2040.

RACE IMPROVEMENT OR EUGENICS, by La Reine Helen Baker, published by Dodd, Mead & Co. 137 small pages, $1.00.

This is an excellent popular presentation of the subject, dealing with the abortion evil, control of offspring, sterilization of the unfit, legislation in the interest of pregnant and parturient women and their offspring, etc. In discussing the feminist movement, it would seem that the complaint that men have not adequately protected married women, is scarcely just. No law can compel the incompetent to be competent nor the criminal to be moral and law-respecting but there is no subject of legislation in which the laws are so absolute, far-reaching and partial, as in those dealing with the duty of support of the wife by the husband. In most states, while the woman loses no property right by marriage, the husband becomes practically a minor so far as title to real property is concerned. In one respect under this heading, we agree thoroughly with the author, however; namely that a man should not be allowed to gamble or speculate to the possible loss of his wife or family. Indeed, we would go farther and urge legislation to check these evils so far as possible, without regard to social state. And, at the risk of appearing to be a misogynist—of which we have never been accused—we would remind the author that a great deal of the speculative evil and of business failures are due to men's ambition to support their families in affluence.

---

BACTERIA, by Dr. Max Schottelius, translated by Staff-Surgeon Herbert Geoghegan, R. N., published by Henry Frowde and Hodder & Stoughton, London, 334 small pages, 10 colored plates and 33 illustrations. $3.50.

While this is a valuable work, the title is somewhat misleading as indicating a systematic consideration of bacteria. It does give a general discussion of bacteriology but it includes protozoic diseases and consists rather in a general treatise on the principles of infection and prophylaxis, immunity, etc. We recommend it to the general practitioner and to the philosophic student. The expert bacteriologist or sanitarian will find little in it with which he is not already familiar and it is not adapted to use as a guide for the detailed study of bacteria.

---

THE THERAPY OF SYPHILIS by Dr. Paul Mulzer of Berlin, translated by A. Newbold. Published by the Rebman Co., New York. 248 pages, cloth, $1.50.

After a brief discussion of the "former nercurial therapy," atoxyl, arsacetin, atoxylate of mercury, hectine, soamin and arsenophenylglycin bring the work up to page 44. From this point on, it is devoted almost solely to the technic and detailed indications and contraindications, dose, etc., of 606.

SECOND ANNUAL REPORT OF THE STATE CHARITIES COMMISSION of Illinois, for the year 1911. Published by the Illinois State Journal Co., State Printers.

This report contains 351 pages and treats in detail of the jails, state hospital, various other institutions and out-door relief. It betokens a spirit of thoroughness and practicality and contains much of interest to students of penology, institutional management, etc., wherever located.

PHYSICAL DIAGNOSIS by Dr. Richard C. Cabot of Boston, published by Wm. Wood & Co., New York; fifth edition, 519 pages, 5 plates, 268 figures, $3.00.

As previously noted, Cabot has an original way of presenting a subject so that, on first opening the book, we have a pleasant disappointment. This does not imply that the heart, lungs and abdominal organs are neglected. On the contrary, the various lesions are exceptionally well presented, with the inclusion of charts of graphic records by instruments of precision. But the face, neck, legs and other regions are included in the book and the term Physical Diagnosis is given the broad interpretation which it deserves.

